# A Rational Explanation of Limited FMD Vaccine Uptake in Endemic Regions

**DOI:** 10.3390/pathogens8040181

**Published:** 2019-10-10

**Authors:** Ashley F. Railey, Thomas L. Marsh

**Affiliations:** 1 Institute for Research and Education to Advance Community Health, College of Medicine, Washington State University, Pullman, WA 99163, USA; 2 Paul G. Allen School for Global Animal Health, Washington State University, Pullman, WA 99163, USA; 3 School of Economic Sciences, Washington State University, Pullman, WA 99163, USA

**Keywords:** Foot-and-mouth disease, game theory, vaccination, vaccines, uncertainty

## Abstract

Vaccination for foot-and-mouth (FMD) disease remains low in parts of Africa despite the existence of vaccines. In East Africa, the presence of multiple virus serotypes and sub-types makes matching a vaccine with the circulating virus type in the field, or providing a high potency vaccine, a challenge. In this paper we use game theory to show that the resulting vaccine uncertainty associated with these vaccination conditions in an endemic setting help explain the low vaccine uptake. We evaluate vaccination for FMD in the context of East Africa due to FMD being endemic in the region, the diversity of FMD virus types, and barriers to implementing other disease control measures, such as controlled movements. We incorporate these conditions into a vaccination game setting and compare the payoffs to those of a traditional vaccination game for seasonal influenza and commercial livestock vaccination in a developed country context. In showing that vaccination provides households with a lower payoff than not vaccinating, our simple game theoretical explanation supports existing evidence calling for improved vaccine quality and efforts to enhance surveillance to provide early information on disease status.

## 1. Introduction

Individuals may decide not to employ disease control measures despite availability and public health recommendations due to individual beliefs and perspectives [1]. Vaccination in human and animal health presents one such case. The decision to vaccinate affects the vaccinated human/animal and contributes to population immunity by reducing the number of susceptible individuals within the community. In contrast, non-vaccinators can ignite and prolong an outbreak depending on who vaccinates, how many remain unvaccinated, and the transmission dynamics of the disease [2]. The decision not to vaccinate may be driven by self-interest, information barriers, and the intention to free ride on the protective immunity of others. For human vaccination, concerns for adverse personal side effects can affect adoption [3], while in non-zoonotic livestock diseases production considerations dominate [4,5]. In contrast, social environments that promote collective action can encourage vaccination [6]. While the dissonance between public health objectives and individual actions is often attributed to these heterogenous individual factors, the disease characteristics, including the severity and timing of infection, likewise affect the decision by influencing the amount of uncertainty involved in the decision process. 

Foot-and-mouth disease (FMD) presents a case whereby the decision to vaccinate involves more than individual deterministic preferences, as uncertainty of vaccine efficacy and vaccine matching also play important roles. FMD is a highly contagious, non-zoonotic viral disease that affects cloven-hoofed animals, including cattle, sheep, and goats [7]. In Africa, FMD-associated production losses amount to around US$2.3 billion annually [8] with the livestock-keeping households directly affected through reduced milk production, lost animal draught power, mortality of young stock, and lost market access [7]. Similar to vaccination for other endemic livestock diseases, vaccination for FMD may help reduce these impacts to improve household income, wealth, and food security [7,9]. This is particularly relevant given livestock keepers in sub-Saharan Africa are disproportionately characterized by extreme poverty [10]. Vaccination for foot-and-mouth disease, along with transnational coordination of policies, contribute to control in South America [11], but the complex spatial and temporal dynamics of multiple viral serotypes with evolving sub-types [12], along with limited infrastructural support to monitor and detect outbreaks within an extensive, pastoral and agro-pastoral system [13] complicates the provision of vaccines in East Africa. The presence of multiple FMD virus types requires either vaccines matched to the circulating virus or sufficiently high potency vaccines to prevent infection [14]. Besides monovalent vaccines, bivalent vaccines (SAT1 and SAT2) and trivalent vaccines (SATs, O, and A) are used in Africa [14]. However, unlike in South Africa, where vaccination accompanies movement restrictions at the wildlife-livestock interface where FMD has been shown to spread [15], in East Africa, inter-herd contacts primarily contribute to transmission [12]. From a household perspective, separating livestock to impose vaccination or other control measures, such as that used in southern Africa, unduly burdens the livelihoods of small-scale livestock owners who rely upon moving animals for market access and grazing [16].

Understanding how the characteristics of a disease and uncertainty of vaccine efficacy influence the individual’s decision to employ disease control measures is important for devising effective control interventions. To this end, we evaluate the decision to vaccinate for FMD in the context of East Africa, where households face persistent disease threat but present with low-vaccination rates. We incorporate these conditions into a traditional vaccination game model by changing the payoff structure to reflect the FMD vaccination scenario. Our theoretical results substantiate empirical findings that attribute vaccination concerns to the quality of the vaccine [17] and extend these findings by showing that these perceptions align with expected behavior given the underlying disease characteristics in the context of East Africa. 

## 2. Materials and Methods

### Vaccination Game Setting

We use game theory to depict the optimal decisions for vaccination strategies with contrasting assumptions about information pertaining to vaccine quality. Game theory has been applied to model various vaccination decisions for the advantage of demonstrating the complex social dynamics that influence vaccination in a theoretical framework [18]. For example, the incorporation of individual behaviors into epidemiological models using the strategic framework of game theory reveals that self-protective behavior influences disease spread in highly contagious epidemics [19]. Similarly, game theory has demonstrated how the pursuit of self-interest changes vaccination uptake overtime [20] and is likely determined by previous experience or exposure to the disease [21]. In contrast to these games that examine differential individual behavior with respect to the evolution of the disease over time, we use a one-shot game to emphasize the role vaccine efficacy and matching play in the decision process. This framework further describes how current circumstances of FMD in East Africa help explain existing findings of low-vaccine uptake [12,17].

Three stylized games outline separate landscapes for the vaccination decision-making process. The first game depicts decisions for an unspecified vaccine with a moderate protection efficacy and cost that closely resembles the vaccination game for seasonal influenza with self-interested individuals [22]. The second game adapts the first to apply to commercial livestock vaccination in a developed country context. The third game then depicts the theoretical decision for uptake of an FMD vaccine in East Africa. Minimal reductions in infection risk characterize the FMD vaccine. For each model we assume a simultaneous-move, one-shot game with homogenous information on the economic costs and losses of disease. The players consist of the individual and others in the community. Each player has two strategies, vaccinate or do not vaccinate, with the payoffs corresponding to the economic costs of each decision given the actions of the other player. While we focus on these three games, other games could be used to motivate the subsequent discussion.

## 3. Results

### 3.1. Strategies and Payoffs

In a traditional vaccination game, non-vaccination represents the Nash equilibrium (Table 1). To portray this decision in a simplified, one-shot game, we assume that ‘free riders’ who do not vaccinate benefit from herd immunity. If the individual vaccinates, then the individual receives protection from the disease, but pays the cost of the vaccine while the community either chooses to also vaccinate (and thus sustain the same conditions) (−3, −3), or free ride on the protective immunity from the vaccinators (−5, 0). The ‘cooperators’ who vaccinate receive a lower payoff. If neither player vaccinates, then both face the risk of infection, but do not suffer the costs of vaccination (−1, −1). In this game, non-vaccination aligns with individual self-interested behavior, especially when adverse vaccine beliefs accompany the decision [23]. To motivate vaccination, either the cost of the vaccine must be sufficiently low or the perceived risk of infection (and consequences thereof) sufficiently high.

For infectious livestock diseases with significant individual costs in commercial agriculture, vaccination is often the dominant individual strategy (Table 2). We assume that a vaccine offers a known probability of protection and the cost of vaccination is less than the costs and losses from an infected herd. If an individual livestock-owning household does not vaccinate the herd, given the payoff structure in Table 2 that corresponds to the incentive structure for vaccinating in commercial settings, the individual will not free ride on the immunity provided by others that vaccinate (−10, −5). If no one vaccinates, the increased likelihood of infection offers the worst payoff for both players (−10, −10). Vaccinating with effective livestock vaccines at market price represents the secure strategy (−5, −5). Regardless of others, vaccinating households incur a small vaccination cost but forego the immediate production losses and longer-term disease implications, including mortality and morbidity risks. Here we have assumed no free riding, for comparison purposes, which could be incorporated, but would not necessarily alter the main points of this paper.

In Table 3, uncertain vaccine efficacy and potency coupled with high infection risk defines the FMD game in an endemic East African setting [12]. Individuals pay for a vaccine with no assurance of the vaccine’s ability to prevent infection. In reality, the individuals have information on the efficacy of past FMD vaccines and control policies, which resulted in limited to no protection against clinical symptoms. If an individual livestock-owning household decides to vaccinate, the individual incurs the cost of the vaccine, the related costs from treating an infected animal, and suffers animal productivity losses, regardless of others’ decisions (−15). If an individual does not vaccinate, then the individual only sustains the costs and losses from the infection (−5). There is no gain to free riding on the vaccination of others. Without the assurance that the vaccine offers a sufficient likelihood of disease protection, the dominate strategy for both players is to not vaccinate (−5, −5). 

### 3.2. Decision Analysis

The high likelihood of infection coupled with the potential production losses attributed to FMD suggest vaccination would address public health concerns. The rapid spread of FMD within dense populations that graze on communal lands makes infection inevitable amongst non-vaccinated animals. Yet, using a vaccine of insufficient potency to prevent or stop the circulating FMD type incurs the additional vaccination cost on top of the lost animal productivity from infection with little to no benefits. Unlike in traditional vaccination games whereby free riding obfuscates vaccination uptake rates, uncertainty towards the protective efficacy of the FMD vaccine reduces incentive to vaccinate in endemic parts of East Africa.

Practically, FMD vaccines need to prevent FMD with a sufficiently high probability. Altering this property then allows the FMD game to resemble that of Table 2. This requires enhancing the payoff of vaccinating by improving vaccine efficacy or reducing infection risk. Subsidizing vaccines to address price constraints may increase uptake, but for FMD, this does not necessarily prevent or stop disease spread. Instead, the assumption of vaccine efficacy may increase vaccination costs if significant barriers to improving vaccine quality exist. Reducing inter-herd contact through restricted animal movements addresses infection risk but the lack of access to markets and constraints on traditional open grazing practices limits implementation in East Africa [24].

## 4. Discussion

A simple game theory model demonstrates how the presence of multiple FMD serotypes and sub-types coupled with the absence of a potent vaccine can make vaccination the least appealing disease control option. This is in contrast to other livestock vaccination programs with one vaccine or even vaccination for seasonal influenza where the vaccine may offer some protection [25]. In East Africa, without the assurance of a certain level of protective efficacy from vaccinating for FMD, vaccination presents an added lost cost on top of the inevitable costs of therapeutic, post-exposure treatments and the associated loss in animal productivity from infection. In effect, without a vaccine matched to the circulating serotype, vaccination is an extraneous expense without any assured benefits. Households in East Africa then reflect rational decision-making when choosing not to vaccinate whereby empirical evidence collected in 2016 and from 2012–2015 found less than five percent of households vaccinated for FMD [12,17].

Improved information about the disease offers an opportunity to reduce the barriers to vaccination. Often, when low vaccination uptake is believed to occur from lack of understanding on the benefits of vaccination, educating local populations on vaccine technology and information campaigns to promote disease control are the preferred interventions [26]. From a theoretical perspective, if low uptake reflects self-interested motives and free riding tendencies, then combating these coordination failures through financial incentives may reduce asymmetries that deter public health objectives for elimination [27]. However, since households currently demonstrate rational behavior by not vaccinating, the challenge to FMD vaccination rests in increasing the likelihood of the vaccine preventing or stopping infection. Similar to influenza vaccination efforts that compile information early to predict the upcoming season’s vaccination needs [28], the diversity of FMD serotypes and sub-types along with the limited use of polyvalent vaccines in East Africa requires sufficient warning to vaccinate. Unlike other approaches to vaccination uptake, encouraging the use of early detection through diagnostic testing or other surveillance measures provides timely information to reduce uncertainty surrounding disease status and potential control options [29] while improved information on disease patterns reduces infection uncertainty.

For vaccination, assessing the disease in context is the first step in deciding whether vaccination is necessary. In some instances, vaccination may not improve individual health outcomes. Addressing individual preferences and adversities towards vaccines incorrectly targets the source of low uptake. Livestock-dependent populations in East Africa already vaccinate for the cattle diseases East Coast Fever and contagious bovine pleuropneumonia when vaccines become available [30,31]. The global eradication of the rinderpest with vaccination further emphasizes the potential for vaccines designed with local communities in mind to address the livestock disease burden [32]. Once FMD vaccines demonstrate improved protection, targeting of susceptible populations to enhance vaccine efficacy [33] and acknowledging the individual dynamic factors that may deter population level elimination [34] come next. Vaccination is not the only disease control measure. For regions with multiple FMD types circulating, identifying patterns in FMD spread posits an additional approach to reducing the challenge of enhancing FMD vaccine efficacy, primarily by predicting vaccine demand instead of necessitating a polyvalent vaccine to address all serotypes [12]. The improved vaccine matching that can occur by understanding the disease spread may then provide spillover benefits, including impacting antibiotic usage and enhancing household expenditures on human health, education, and food [9].

The strategies presented here represent a simplified version of the vaccination game for complex diseases with ineffective or non-existent vaccines. The information is particularly relevant given current global challenges to create FMD vaccines specific to East Africa [35]. The framework developed through the game theoretical model encourages those individuals seeking to define the demand for FMD vaccines to consider the costs of a low-quality vaccine and lack of quality assurance. Extending our theoretical analysis to a dynamic setting with repeated interactions and improved vaccine quality would provide an initial understanding on the stability of vaccination uptake if vaccination rates increased [20]. However, first understanding how to improve the efficacy of livestock vaccines in the field and identifying additional critical decision points to ensuring the optimal provision of health technologies will help increase the effectiveness of livestock inputs while enhancing returns to household wellbeing. Once FMD vaccines for East Africa ensure households may receive some protection from FMD, we can then perform cost-effectiveness analyses to evaluate the impact across the diverse livestock systems, similar to studies evaluating current FMD vaccination practices elsewhere [24].

## Figures and Tables

**Table 1:** Vaccine is effective.

Individual/Others	Vaccinate	Do not Vaccinate
Vaccinate	(−3, −3)	(−5, 0)
Do not vaccinate	(0, −5)	(−1, −1)

**Table 2:** Vaccine is effective, long-term disease complications.

Individual/Others	Vaccinate	Do not Vaccinate
Vaccinate	(−5, −5)	(−5, −10)
Do not vaccinate	(−10, −5)	(−10, −10)

**Table 3:** Vaccine is not effective.

Individual/Others	Vaccinate	Do not Vaccinate
Vaccinate	(−15, −15)	(−15, −5)
Do not vaccinate	(−5, −15)	(−5, −5)
